# Real-time color flow mapping of ultrasound microrobots

**DOI:** 10.1126/sciadv.adt8887

**Published:** 2025-07-18

**Authors:** Cornel Dillinger, Ahilan Rasaiah, Abigail Vogel, Chaimae Bahou, Katia Monastyrskaya, Ali Hashemi Gheinani, Daniel Ahmed

**Affiliations:** ^1^ Acoustic Robotics Systems Lab, Department of Mechanical and Process Engineering, ETH Zurich, Zurich, Switzerland.; ^2^ Functional Urology Research Group, Department for Biomedical Research, University of Bern, Bern, Switzerland.; ^3^ Functional Urology, Department of Urology, Inselspital, Bern University Hospital, University of Bern, Bern, Switzerland.

## Abstract

Visualization and tracking of microrobots in real time pose key challenges for surgical microrobotic systems, as existing imaging modalities like magnetic resonance imaging, computed tomography, and x-ray are unable to monitor microscale items with real-time resolution. Ultrasound imaging–guided drug administration represents a remarkable advancement in this respect, offering real-time visual feedback on invasive medical procedures. However, ultrasound imaging still faces substantial inherent limitations in spatial resolution and signal attenuation, which hinder extending this method to microrobot visualization. Here, we introduce an approach for visualizing individual microrobots in real time with color flow mapping ultrasound imaging based on acoustically induced structural oscillations of the microrobot generating a pseudo-Doppler signal. This approach enables the simultaneous localization and activation of bubble-based microrobots using two ultrasound sources operating at distinct frequency bandwidths. Our successful capture of microrobots measuring 60 to 80 micrometers in diameter reveals the potential of real-time ultrasonic imaging at the microscale.

## Teaser

Advanced ultrasound imaging enables real-time, microscale visualization—a critical breakthrough for therapeutic microrobots.

## INTRODUCTION

Microrobots capable of being wirelessly manipulated within the human body can revolutionize the future of medicine and offer innovative solutions in noninvasive therapeutics and surgical procedures (*1*, *2*). In particular, these microscale medical assistants can enable targeted therapeutics, the delivery of drugs, genes, or cells to hard-to-reach and sensitive locations (*3*–*10*). For all their capabilities, however, a major limitation in current procedures is the absence of real-time visual feedback on the microrobot’s position in deep-seated tissues, which is crucial for their effective use in medical treatments (*11*). Various biomedical imaging modalities such as magnetic resonance imaging, x-ray and computed tomography scanning, ultrasound imaging, photoacoustic imaging, magnetic particle imaging, and positron emission tomography are being explored for microrobot imaging (*12*–*18*). However, each of these techniques faces its own set of challenges, such as low resolution and contrast (ultrasound imaging), risks of ionizing radiation (x-ray and computed tomography), the need for high-resolution real-time data (magnetic resonance imaging), limited penetration depth (photoacoustic imaging), and the inability to simultaneously visualize both the microrobot and surrounding environment (magnetic particle imaging and positron emission tomography). These limitations highlight the need for advanced imaging solutions capable of real-time, high-resolution visualization of microrobots within the human body (*19*).

Ultrasound imaging, with its widespread clinical use, real-time capability, and deep tissue penetration (up to 25 cm), stands as a suitable candidate for microrobot imaging (*19*). Gas-filled ultrasound contrast agents can enhance the spatial resolution of ultrasound imaging by exploiting the high acoustic contrast at gas-liquid interfaces, which provides them with high echogenicity (reflection of acoustic waves) compared to surrounding tissue (*20*, *21*). This improves microscale object detectability in the commonly applied brightness mode (B-mode), in which two-dimensional grayscale images are generated from the echo intensities of in-tissue reflected ultrasound waves. Recently, magnetomotive ultrasound imaging concepts have been adapted for the real-time visualization of magnetic field–responsive microrobots. This imaging mode exploits the Doppler effect to detect shifts in ultrasound wave frequency as the waves reflect off moving objects, thereby providing information on their velocity and direction of motion. These data are then overlaid on a conventional B-mode image in the form of a colored flow map, also termed color flow mapping (CFM)-mode imaging. Current applications include discerning the motion of a magnetic nanoparticle swarm and detecting acoustic phase shifts in ultrasound signals linked to the controlled vibration of a magnetic microrobot (*22*–*24*). These studies demonstrate how CFM-mode imaging can overcome resolution and sensitivity limitations of ultrasound imaging for magnetic field–driven microrobots. Nevertheless, the efficacy of magnetomotive ultrasound imaging remains constrained by the fact that microrobots are constructed from materials having limited acoustic contrast with adjacent tissue; accordingly, the visualization has limited resolution and sensitivity, and advanced signal processing algorithms are required (*25*, *26*). Recent work by Kim *et al.* (*27*) has shown promising results in using magneto-gas vesicles to increase acoustic contrast in magnetic microagents. However, this technique has primarily been explored for tissue stiffness diagnosis, where only small agent displacements are assessed.

In this study, we present real-time CFM-mode ultrasound imaging of individual high acoustic contrast bubble-based microrobots (~73 μm in diameter). The imaging concept relies on acoustically stimulated oscillation of the microrobots generated by encapsulated microbubbles that serve as both the propulsion unit and contrast agent. Stationary but oscillating microrobots are detected by the imaging ultrasound as pseudo-moving objects, leading to random frequency shifts in the reflected ultrasound signals, referred to as pseudo-Doppler frequency shifts (*28*–*30*). The same effect is also observed in microrobots that are in motion. Notably, such bubble-based microrobots exhibit remarkable propulsion capabilities, driven by simple piezoelectric-driven acoustic stimulation in the liquid environment (*31*–*36*). We simultaneously harness propulsion while using the strong acoustic contrast and emissions of the stimulated microrobots to enable real-time detection using a conventional ultrasound imaging system (imaging frequency $f_{\text{US}}=4.0\;\text{to}\;16.0\;\text{MHz}$ ). We demonstrate and characterize an imaging method that visualizes individual microrobots in real time during translational motion and detects them from various angles within deep phantom tissue (up to 10 cm) and in an ex vivo mouse bladder model. Together, this work showcases the potential dual functionality of acoustic microrobots: wireless propulsion with real-time ultrasound imaging.

## RESULTS

### Imaging concept

**Fig. 1.**
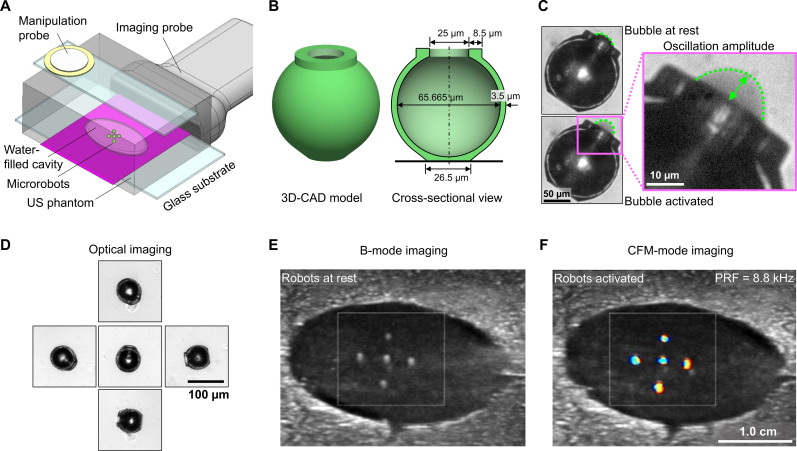
CFM imaging concept. (**A**) Schematic of the experimental setup with simultaneous imaging of the same plane, optically through the glass substrate below and by ultrasound imaging from the side. (**B**) 3D-CAD model, cross-sectional view, and dimensions of the bubble-based acoustic microrobot. (**C**) When actuated with an externally applied acoustic field with frequency f=100.0kHz and amplitude $V_{\text{pp}}=7.0\;V$ , an entrapped microbubble is stimulated to oscillate at large amplitudes. (**D**) Optical imaging of microrobots in cross formation. (**E** and **F**) Ultrasound imaging of microrobots using B-mode (E) and CFM mode (F) in a conventional mobile ultrasound imaging system.

Our demonstration of the microscale ultrasound imaging concept used five bubble-based acoustic microrobots arranged in a cross formation on a polydimethylsiloxane (PDMS) spin-coated glass slide. This glass slide was placed underneath an agar-based phantom block with an elliptical concavity on its bottom side, enclosing a cavity filled with deionized (DI) water. Using this setup, we can simultaneously observe single microrobots optically from below through the objective of an inverted microscope and ultrasonically from the side using a linear array ultrasound imaging probe. In this configuration, both optical and ultrasound imaging methods visualize the plane wherein the microrobots are positioned (Fig. 1A).

The 3D-printed bubble-based microrobots consist of hollow polymeric spheres with an outer diameter of $D_{\text{outer}}\approx 72.6\;\mathrm{\mu m}$ , a minimal wall thickness of W≈3.5μm , and a spherical orifice on one side with a diameter of A≈25.0μm (Fig. 1B). The microrobot’s dimension and shape were selected according to two key factors: (i) generating radial oscillations enabling 360°-CFM imaging and (ii) reducing interferences of the stimulating and imaging acoustic fields. Designed encapsulation of spherical microbubbles, with d≈65.6μm in diameter, enabled strong radial oscillation at their theoretical resonance frequency, which is calculated to be around f≈100.0kHz (*37*). Moreover, this stimulation frequency was matched with well-resonating frequency peaks of our stimulation probe ( f=95.0 to 108.0kHz ), enabling robust microrobot actuation and a demonstration of 180°-CFM imaging through tissue-like ultrasound phantom blocks of thicknesses t≈2.0 to 4.0cm . Notably, also microrobots half the size, i.e., ~37 μm in diameter, have been successfully imaged using CFM mode and the same stimulation frequency f=100.0kHz (fig. S1), suggesting that for CFM-mode imaging, resonance frequency stimulation is nonessential. Because two ultrasound sources were operating within the same domain in this study, potential signal interferences were expected. Hence, to minimize imaging disturbances such as B-mode brightness fluctuations caused by acoustic and electromagnetic interferences at stimulation frequencies of f≥2.0MHz (see fig. S2), we found the stimulation frequency of f≈100.0kHz—one order of magnitude lower than our imaging frequencies ( $f_{\text{US}}=4.0\;\text{to}\;16.0\;\text{MHz}$)—helpful to mitigate imaging signal interferences (*38*, *39*).

After the 3D-printing and development process, microrobots were dried and underwent hydrophobic silane coating to stabilize entrapped microbubbles (see Materials and Methods). Subsequently, we positioned five of the microrobots in cross formation on the PDMS-coated glass slide. Because of the strong adhesion of the microrobots to the hydrophobic PDMS layer, attributed to hydrophobic interaction forces enhanced by their silane coating, the robot-containing glass slide could be flipped upside down to enclose the water-filled cavity of the ultrasound phantom (*40*, *41*). After enclosing the cavity, the phantom-glass slide complex was flipped back by 180° and positioned on the microscope stage for investigation. When both microrobot positioning and bubble entrapment were optically detected, we coupled the imaging (side) and manipulation (top) ultrasound probes to the phantom using ultrasonic coupling gel. The manipulation probe compromises a glass slide, onto which a piezoelectric transducer disc is attached, driven by a function generator and a signal amplifier.

When the manipulation probe was stimulated with a sinusoidal frequency of around f=95.0 to 108.0kHz , the entrapped microbubbles exhibited strong oscillation amplitudes up to ε≈10μm (Fig. 1C). Such oscillation introduces acoustic streaming in the form of two counter-rotating vortices with a common backward jetting stream at the center of the orifice, generating propulsion in the opposite direction, a phenomenon shown in previous work (*42*–*44*). The sticky layer of PDMS on the bottom slide held the robots in position, allowing for continuous characterization and investigation of the imaging capability (Fig. 1D). In the water-filled elliptical chamber of the phantom, we visualized the microrobots in regular B-mode (Fig. 1E) and then in the CFM mode of the ultrasound imaging system (Fig. 1F), which provided improved contrast and visibility, consistent with earlier findings on ultrasound contrast agents (*28*, *45*). In CFM mode, the ultrasound imaging system gathers multiple signals (reflected sound waves) across a predetermined region of interest and converts these data into color tones on the basis of the detected Doppler frequency shift, which indicates the direction and velocity of the detected object. Specifically, a blue tone denotes motion away, whereas a red tone indicates motion toward the imaging probe. In our case, it is likely that the moving object being detected is the oscillating robot-liquid interface ( ∼f=100.0kHz ), which moves back and forth with respect to the imaging probe. This can result in varying Doppler frequency shifts generated in subsequent Doppler pulses [emitted at pulse repetition frequency (PRF)], which are visualized as color-flickering CFM-mode images. In the literature, it has been reported that such microbubble oscillations can lead to a loss of correlation in an imaging system’s signal analysis, resulting in so-called pseudo-Doppler signals that depict the microbubbles as random, mosaic-colored dots (*28*, *29*).

Notably, when adjusting the ultrasound imaging system’s PRF (pulse values being $\begin{array}{c} f_{\text{PRF}}=10.0,8.8,8.0,7.0,6.7,\;\ldots ,1.8,1.5,1.2,1.0, \end{array}$  and 0.5kHz) while stimulating the microrobots with an acoustic field at precisely f=100.0kHz , no CFM signal was detected whenever $f\;/\;f_{\text{PRF}}$ resulted in an integer. Conversely, when the stimulating frequency was not an integer multiple of the PRF (e.g., $f_{\text{PRF}}=8.8\;\text{kHz}$ ), the microrobots exhibited a CFM signal, observed as a red-to-blue fluctuation (table S1). This finding supports our understanding that the detecting Doppler pulse (emitted at PRF rate) and the oscillating microrobot can be in phase with each other, meaning that while the microbubble oscillates back and forth, the detecting Doppler pulse only interacts with and hence “sees” the robot’s interface as being stationary. Moreover, this finding further indicates that in our experiments, the microbubbles primarily respond linearly—oscillating and generating signals—at the same acoustic frequency as their stimulation frequency, f=100.0kHz.

### Imaging characterization

**Fig. 2.**
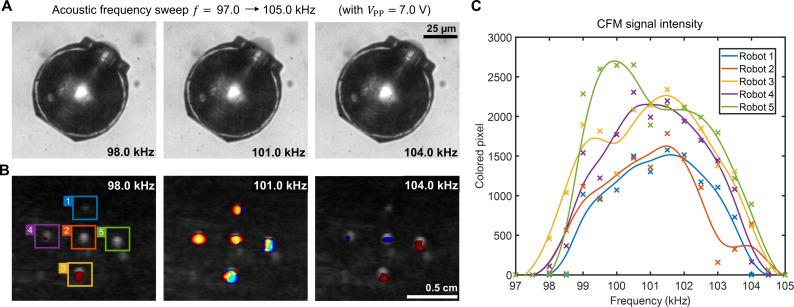
Acoustic frequency sweep. (**A**) When stimulated with acoustic frequencies in the range of f=97.0 to 105.0kHz , the largest microbubble oscillation amplitudes were observed near the manipulation probe’s resonance frequency band of $f_{\text{res}}\approx 100.0\;\text{to}\;101.5\;\text{kHz}$ . (**B**) Similarly, the most pronounced CFM signals were captured at f≈100.0 to 101.5kHz , indicated by the brightly colored dots representing the microrobots placed in cross formation. (**C**) Plot of the average number of colored pixels in each frame of recorded CFM-mode videos versus applied frequency. Imaging analysis was performed by a custom-coded pixel counter algorithm.

To further understand the CFM-mode imaging concept, we characterized the entrapped microbubble’s response to an acoustic frequency sweep around its main stimulation frequency bandwidth ( f=100.0 to 101.5kHz ). We captured the resultant microbubble actuation using both a high-speed camera and the ultrasound imaging system (movie S1). As anticipated, the microbubble’s oscillation amplitude reached its peak when the stimulating acoustic field approached the manipulation probe’s resonance frequency band $f_{\text{res}}\approx 100.0\;\text{to}\;101.5\;\text{kHz}$ (Fig. 2A). At these frequencies, the CFM signals captured were most prominent, as shown by the highest number of colored pixels in the sonograms and in the plot of Fig. 2 (B and C), indicating the correspondence of the CFM signal with the microbubble’s actuation**.**

**Fig. 3.**
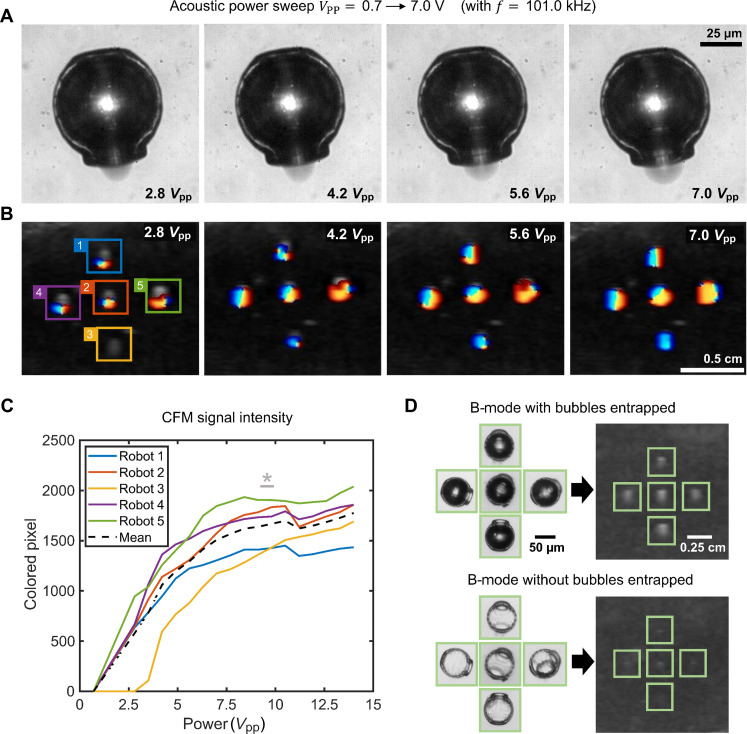
Acoustic power sweep. Microrobots exposed to increasing *V*_pp_ amplitudes ranging from $V_{\text{pp}}=0.7\;\text{to}\;14.0\;V$ were imaged using (**A**) optical microscopy and (**B**) CFM-mode ultrasound imaging. (**C**) Plot of the average number of colored pixels in each frame of recorded CFM-mode videos versus applied power. The asterisk (*) indicates a set of linear interpolated (missing) data points at the value of $V_{\text{pp}}=9.1\;V$ . (**D**) Optical and B-mode imaging of microrobots before (top) and after (bottom) application of high power amplitudes ( $V_{\text{pp}}>50.0\;V$ ). Using such high amplitudes, the entrapped microbubbles often vanish, making ultrasonic detection challenging.

**Fig. 4.**
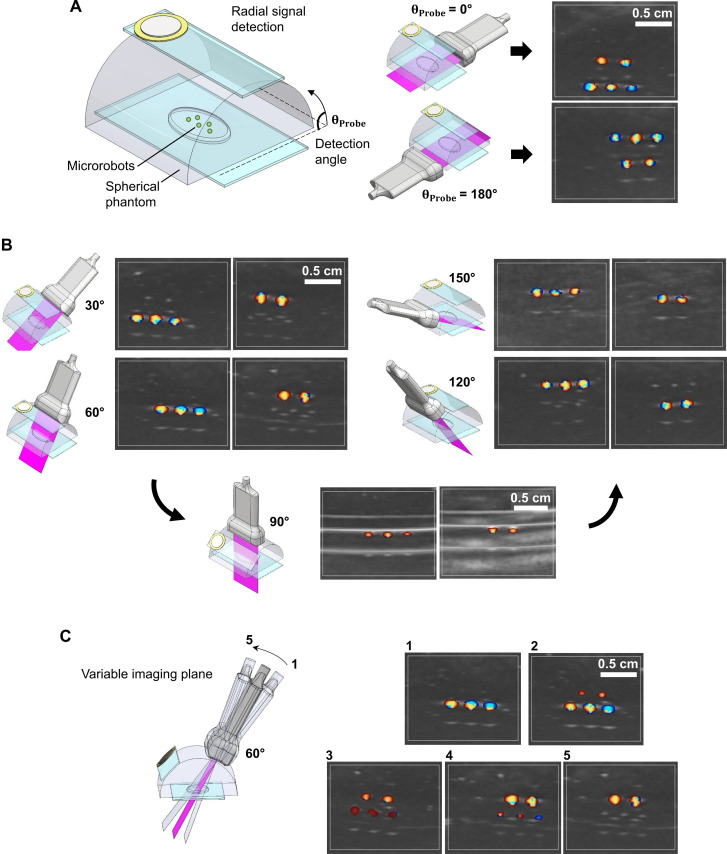
Radial signal detection. (**A**) Schematic showing the placement of five microrobots when analyzing microrobot detectability from various imaging angles. Both rows of microrobots could be imaged in plane with the glass substrate when the ultrasound imaging probe was held at $\theta_{\text{Probe}}=0\;{}^{\circ}$ or 180° . (**B**) Successful imaging of microrobots at imaging angles ranging from $\theta_{\text{Probe}}=30\;{}^{\circ}\text{to}\;150\;{}^{\circ}$ . In these cases, the surface of the glass substrate does not align with the ultrasound imaging plane. Therefore, the two rows of microrobots were separately visualized by partially rotating the imaging probe around the point of contact with the ultrasound phantom, as illustrated in (**C**).

Next, we conducted acoustic amplitude sweep experiments. When we exposed microrobots to an acoustic field of f=101.0kHz and peak-to-peak voltage ( $V_{\text{pp}}$ ) amplitudes of $V_{\text{pp}}=0.7\;\text{to}\;14.0\;V$ , we observed that increasing the voltage led to a larger oscillation amplitude, which was visualized in both optical and CFM-mode ultrasound imaging modalities (Fig. 3, A and B, and movie S2). When plotting the total number of colored pixels in relation to applied *V*_pp_ amplitudes, a buckling was observed at $V_{\text{pp}}\approx 7.0\;V$ , followed by a plateau in signal response (Fig. 3C). In our setup, operating microrobots in the power range $V_{\text{pp}}<40.0\;V$ has proven sustainable for investigations spanning several minutes. Applied amplitudes exceeding $V_{\text{pp}}\approx 50.0\;V$ often caused microbubbles to collapse, as shown in fig. S3. In Fig. 3D, we showcase the importance of continuous microbubble entrapment for ultrasonic detection of the microrobots. Without any microbubble trapped, no CFM signal is generated; furthermore, in B-mode (imaging frequency $f_{\text{US}}=10.9\;\text{to}\;14.0\;\text{MHz}$ ), none of the five microrobots are distinguishable. Furthermore, to verify that a robot’s orifice orientation is not crucial for imaging, we positioned four microrobots in various orientations relative to the imaging probe (fig. S4). We detected no correlation between the color patterns in the CFM signal and the relative orientation of a microrobot’s orifice. Further analysis of Fig. 3 revealed that the CFM signal intensity—quantified as the number of colored pixels—can vary among microrobots printed with identical dimensions depending on their placement within the detecting ultrasound beam. For instance, the microrobots in the middle row of Fig. 3B displayed markedly higher signal intensities compared to those positioned at the top and bottom, with variations reaching up to ~300 pixels (Fig. 3C). This observation is consistent with our understanding that the detecting ultrasound beam (emitting transducer surface: 49 mm by 10 mm) interferes well with the actuated robots of the middle row and less with the robots on the top and bottom. This finding supported the next experiment resulting in the data in Fig. 4, which not only demonstrates 180° robot detectability but also shows that by varying the imaging probe’s active surface contact angle (ultrasound beam direction), we were able to gradually distinguish between two separate rows of positioned robots.

A vital aspect of real-time microrobot imaging within the human body is the ability to effectively detect the microrobots from any angle and over the distance required in the given situation. In this regard, ultrasound imaging benefits from its simple and portable nature, which allows medical personnel to adapt and optimize the imaging procedure according to need. In Fig. 4, we demonstrate the detection of our bubble-based acoustic microrobots from various imaging angles. For this experiment, we designed a tissue phantom with a curved surface that allowed us to assess the imaging probe’s ability to visualize microrobots over a 180° perspective. Inside the water-filled chamber of the phantom, five microrobots were positioned in two rows with two in one row and three in the other. As in previous experiments, the simultaneous visualization of all five microrobots was facilitated by initially holding the imaging probe in a parallel plane to the glass substrate at angles $\theta_{\text{Probe}}=0\;{}^{\circ}$ and 180° (Fig. 4A). We then moved the imaging probe upward toward imaging angles of 30° , 60° , and 90° , during which the microrobots remained detectable, exhibiting consistent signal intensity. We attribute this phenomenon to the radial oscillation patterns of the activated microbubbles entrapped within the microrobots. Further manual (hand-held) guidance of the imaging probe demonstrated that the oscillation signal could be detected from any imaging angle (Fig. 4B). However, when the imaging plane, indicated in purple, deviated from the planes at $\theta_{\text{Probe}}=0\;{}^{\circ}$ and 180° , simultaneous visualization of all five microrobots became unattainable. This is expected as the imaging ultrasonic beam has limited height and depicts only objects within its imaging plane. When the imaging angle is adjusted away from the parallel-to-ground plane, the imaging plane interferes only with one of the rows of microrobots. In Fig. 4C, we demonstrate that at $\theta_{\text{Probe}}=60\;{}^{\circ}$ , it is feasible to adjust the imaging plane to display both rows; however, imaging both rows with full intensity at the same time remains unachievable (see movie S3). This result highlights the challenge of locating the correct imaging plane when the objects to be detected are of microscale dimensions.

### Deep tissue visualization

**Fig. 5.**
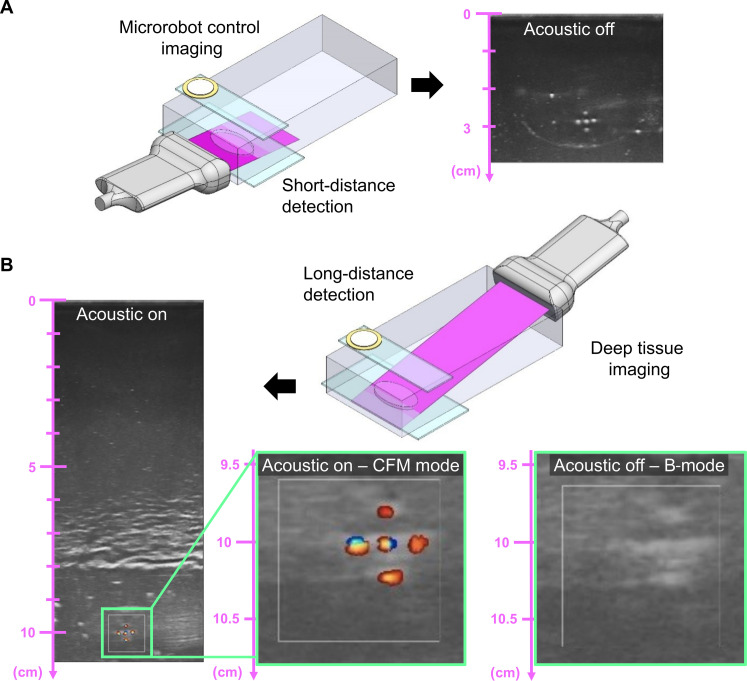
Deep tissue visualization. An elongated ultrasound tissue phantom was used to test microrobot detectability at different depths. (**A**) Visualization in B-mode at the short depth of approximately D=3cm . (**B**) Imaging of microrobots from the opposite side over a distance of approximately D=10cm . In the absence of acoustic stimulation (B-mode), detecting the microrobots is challenging. However, when acoustics are enabled, the CFM signal clearly detects all five microrobots.

Real-time high-resolution visualization of microrobotic therapeutics in deep-seated tissue remains a challenging task. In Fig. 5, we present real-time CFM-based ultrasound imaging of our microrobots in agar-based tissue phantoms, covering depths up to D=10cm . First, we validated the presence of five microrobots in cross formation from an imaging distance of about D=3cm (Fig. 5A). After confirming the presence of the trapped microbubbles under the microscope, we repositioned the imaging probe to the opposite side of the tissue phantom and began detecting signals from the microrobots at distances as far as D=10cm . Scanning the DI water–filled chamber to detect the microrobots includes the consideration of the ultrasound beam’s refraction at the tissue-water interface according to Snell’s law (*46*). When ultrasound passes through an interface between two media with varying propagation speeds, the sound beam, i.e., the imaging plane, becomes bent, leading to a nonintuitive imaging angle of the probe required to find the desired imaging plane. The usage of the CFM imaging mode helped to identify the microrobots in deep tissue situations as illustrated in Fig. 5B. When the acoustic field was stimulated at f=101.0kHz and $V_{\text{pp}}=21.0\;V$ , a clear visible signal of each microrobot was detected. However, in the absence of an acoustic stimulus, the location of the microrobots was hard to identify because only a weak, grayish blurred region indicated their presence (movie S4). This result emphasizes the benefits of using CFM-mode in addition to conventional B-mode imaging when detecting microscale objects equipped with microbubbles.

### Real-time motion imaging

**Fig. 6.**
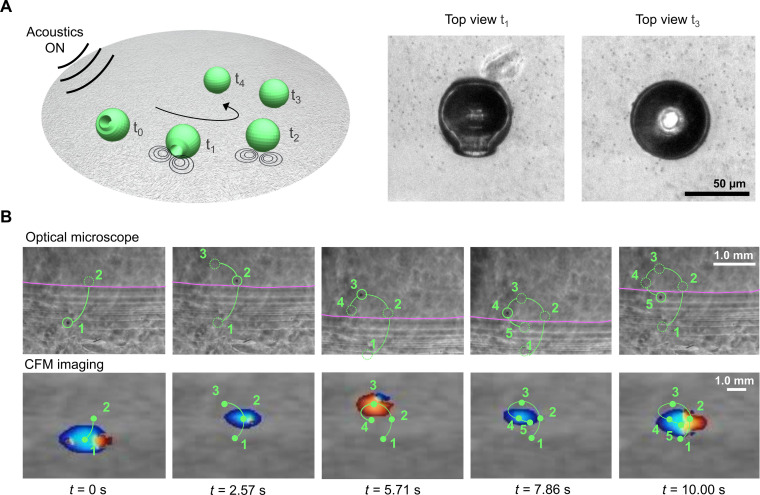
Real-time motion imaging. (**A**) When acoustics are activated, the bubble-based microrobot starts to generate acoustic streaming and reorients itself, aligning its orifice perpendicular to the substrate before undergoing spherical motion patterns. (**B**) Image sequence depicting simultaneous capturing of a swimming microrobot under an optical microscope and ultrasound imaging in CFM mode. Acoustic stimulation with f=101.0kHz and $V_{\text{pp}}=8.4\;V$ resulted in translational motion along a spherical trajectory recorded by both imaging modalities.

In this section, we aim to emphasize one of the biggest advantages of ultrasound imaging—the real-time representation of live data. For the design of precise microrobotic-assisted therapeutics such as the application in targeted drug, gene, or cell delivery, microsurgery, or diagnostic sensing, continuous real-time feedback on the microrobots’ performance is essential. This involves monitoring the microrobots’ movement toward the designated site, verifying the successful execution of their task, and confirming that the treatment has not exacerbated the situation. In this context, we showcase the successful real-time detection of our acoustically driven bubble-based microrobots in translational motion. To enable the free movement of the microrobots in this experiment, we replaced the sticky PDMS spin-coated glass substrate with a noncoated smooth glass slide. When a bubble-based microrobot is activated on such a smooth substrate and immersed in an Newtonian fluid (DI water), it starts to generate acoustic streaming and reorients itself, aligning its orifice perpendicular to the substrate before undergoing spherical motion patterns (Fig. 6A) (*43*). Notably, it has been observed that the behavior of this class of microrobots can vary depending on their fluid surroundings: In Newtonian fluids, their openings tend to orient perpendicular to a rigid surface because of the secondary Bjerknes effect, whereas in non-Newtonian fluids, they may adopt a parallel orientation (*33*). Nonetheless, the ultrasound CFM imaging performance remains consistently independent of the microrobots’ orientation.

A secondary effect, supported by the smooth glass substrate, resulted in the microrobots’ immediate movement toward the cavity wall as soon as the imaging probe was positioned on the tissue phantom. Upon entering the DI water–filled chamber, the ultrasonic waves from the imaging probe induced acoustic bulk streaming (see also fig. S5), known as Eckart streaming (*47*). In prior experiments, this effect remained negligible because of the substantial adherence generated by the PDMS-coated glass substrate, which held the microrobots in position. Nevertheless, the acoustic bulk streaming, flowing in the direction away from the imaging probe, resulted in the microrobots being pushed toward the wall of the cavity situated on the opposite side from imaging probe. This represents the initial position of the microrobot depicted in the first frame of Fig. 6B. We then applied an acoustic stimulation of f=101.0kHz and $V_{\text{pp}}=8.4\;V$ on the manipulation probe to actuate the microrobot and set it into motion. This effective wireless acoustic propulsion principle has been demonstrated in various works (*32*, *33*). In our specific scenario, the propulsive force facilitated the microrobot’s detachment from the wall, leading to characteristic trochoidal motion patterns (*43*). After completing two spherical trajectories, the microrobot returned to the wall where the acoustic stimulation was turned off. We simultaneously recorded the microrobot’s motion with a charge-coupled device camera and the ultrasound imaging system in CFM mode, therefore demonstrating the capability to real-time image the motion of bubble-based microrobots (Fig. 6B and movies S5 and S6). In an additional experiment, using a 6-μm flow tracer immersed in the robots’ aquatic environment, we verified the situation where stationary microrobots are exposed to a background flow, i.e., the previously introduced steady-state bulk streaming. Because of acoustic waves reflected from the 6-μm flow tracers, the CFM mode correctly detected steady streaming; however, it did not prevent the visualization of the microrobots (fig. S3). In conclusion of this experiment, we realized that the operating imaging probe can introduce streaming effects into the aquatic environment of the microrobots. However, because of the powerful bubble-based propulsion used, the microrobots can overcome this environmental stress and remain visible, even in the presence of background flow.

### Visualization of a drug delivery mechanism

**Fig. 7.**
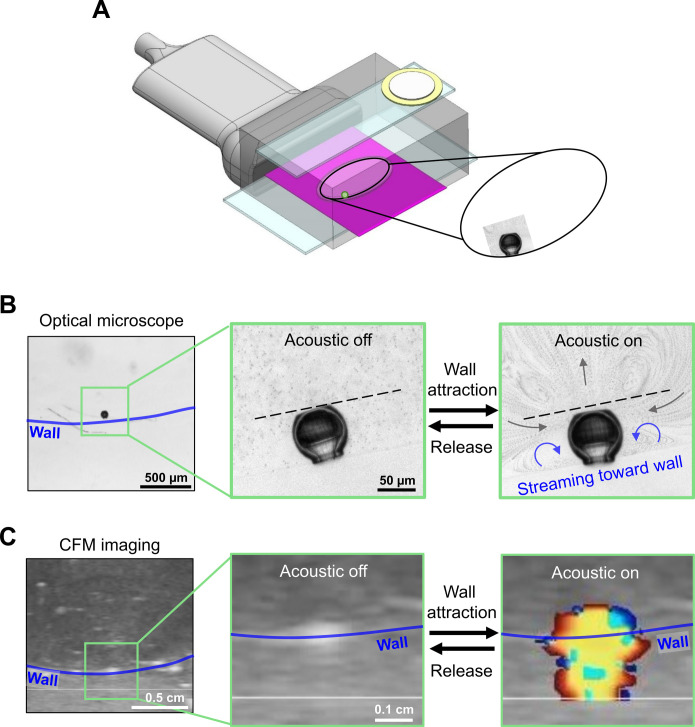
Real-time ultrasound imaging feedback in a drug delivery mechanism. (**A**) Schematic illustrating the direction of a microrobot toward the wall of the cavity in the ultrasound tissue phantom. (**B**) Upon reaching the wall, the microrobot orients its orifice toward the wall, introduced by acoustic stimulation with f=103.4kHz and $V_{\text{pp}}=35.0\;V$ . Activating and deactivating the acoustics control the microrobot’s attraction to and release from the wall, accompanied by acoustic streaming directed toward the wall. (**C**) CFM imaging reveals the microrobot’s actuation at the wall when acoustics are turned on.

In addition to real-time imaging capabilities, we demonstrate in Fig. 7 a potential drug delivery task and how it could be visualized using our proposed CFM imaging approach. In Fig. 7A, we illustrate how a microrobot orients its orifice and is attracted toward the cavity’s wall governed by the secondary Bjerknes force effect, introduced by oscillating bubbles near rigid walls (*48*). A bubble-based microrobot can be manipulated to adhere to the wall and release from it once the acoustics are turned off (Fig. 7, B and C). This controllable adherence to the wall is accompanied by acoustic microbubble streaming toward the wall (Fig. 7B and movie S7), which can be used to direct dissolved drugs toward targeted regions in the cavity’s wall. Furthermore, seeing the resultant CFM-mode signal of such an event (Fig. 7C), we envision that our real-time visualized microrobots can accomplish targeted tasks to specific locations.

### Ex vivo imaging of microrobots in the mouse bladder

**Fig. 8.**
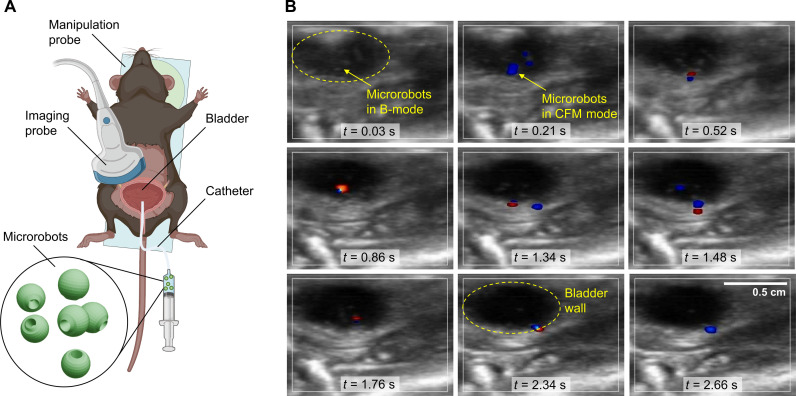
CFM-mode ultrasound imaging of microrobots in the mouse bladder ex vivo. (**A**) Schematic illustration of the ex vivo experiment. The mouse model was bedded on an inverted manipulation probe. With the ultrasound imaging probe applied from the top, CFM signals of swimming microrobots were captured. (**B**) After the injection of microrobots, grayish blurred dots indicated the presence of bubble-based acoustic microrobots in B-mode imaging. However, distinct CFM signals were captured from within the dark-colored bladder, visualizing the microrobots’ presence and motion toward the bottom bladder wall when manipulated by acoustic stimulation of f=99.5 to 101.5kHz and $V_{\text{pp}}=45.0\;V$.

We continued to validate the real-time CFM-mode imaging concept in a physiologically relevant environment, namely, in an ex vivo mouse bladder model. In the experiment, the mouse was positioned on our inverted manipulation probe (piezo-adhered glass slide) and imaged from the top using the imaging probe, with ultrasound coupling gel applied between the probes and the soft tissue for acoustic impedance coupling (Fig. 8A). We then injected a batch of 400 microrobots, originally immersed in 200 μl of sterile water, by delivering ~100 μl of the suspension into the mouse bladder via a catheter. Under acoustic stimulation of f=99.5 to 101.5kHz , $V_{\text{pp}}=45.0\;V$ , and simultaneous ultrasound imaging from the top, we captured ultrasound signals of individual swimming microrobots. Initially, the swarm of microrobots was challenging to observe with B-mode signals only; however, distinct CFM signals from within the homogeneous dark-colored fluid of the bladder indicated the microrobot’s presence and movement toward the bottom bladder wall (Fig. 8B and movie S8). As observed in the previous experimental results of Fig. 6 (real-time motion imaging) and Fig. 7 (drug delivery mechanism), the microrobots were attracted toward the cavity wall, i.e., the bottom bladder wall, where CFM signals were detected at the interface. This result confirms the acoustic manipulation of the microrobots in the ex vivo experiment, as bubble-based robots are expected to ascend to the top bladder wall because of buoyancy forces. Whether the dominant manipulation effect in the ex vivo experiment arises from acoustic streaming generated by the microrobots, acoustic bulk streaming (Eckart streaming) induced by the imaging probe, bubble-induced wall-attractive secondary Bjerknes forces, or a combination of these mechanisms remains to be explored (*47*, *48*). Overall, the real-time imaging in the ex vivo mouse model was achieved by setting the ultrasound system’s wall filter to its maximum value and fine-tuning the signal gain to low-to-moderate levels (55 of 255), effectively suppressing artifact CFM signals within the soft tissue.

## DISCUSSION

Using the presented imaging method for acoustic bubble-based microrobots, the inherent advantages of ultrasound imaging—such as cost-effective and portable equipment, real-time data representation, and deep tissue penetration—are enhanced by the effective visualization of micro-objects equipped with high acoustic contrast. Entrapped microbubbles simultaneously act as the propulsion unit driving the microrobots and as a contrast agent enhancing their detectability. The imaging concept leverages the improved sensitivity of the CFM mode in conventional ultrasound imaging systems to real-time visualize stationary and moving micro-objects, e.g., microrobots. Recent advances have used ultrasound Doppler methods to detect robot movements, aiming to overcome the resolution deficits of B-mode imaging; however, these approaches are challenged by low velocities of external field–driven microscale motion. With this work, we address these limitations by demonstrating kilohertz-frequency actuation of 3D-printed bubble-based microrobots, leveraging their inherent acoustic contrast and high-frequency motion to enhance visualization via CFM-mode ultrasound imaging. Given the challenges posed by depth in ultrasound imaging, it is equally important to consider how the structural complexity and varying properties of biological tissues affect the imaging performance of microrobot-assisted applications. As acoustic waves propagate through the body, their amplitude and intensity decrease because of absorption, reflection, and scattering. These losses result from factors such as viscosity, heat conduction, and molecular energy exchange, which can affect image quality and signal penetration (*49*). Further signal attenuation occurs when a wave transitions between tissues with different acoustic impedances, resulting in an impedance mismatch that leads to partial wave reflection (*50*). However, ultrasound imaging, including CFM-mode imaging, benefits from high water content in human soft tissue and low impedance mismatches along the penetration path to the target organs (*51*). This reduces acoustic attenuation and reflections, allowing for efficient wave transmission. As a result, ultrasound CFM imaging remains effective for real-time microrobot visualization in deep tissue because of maintained signal intensity.

We emphasize promising application of our microrobots in fluidic cavities lined with mucosal tissue—such as the stomach, intestines, and as demonstrated in bladders—where local delivery methods (e.g., needle injection, endoscopic deployment, or controlled capsule release) can be effectively used (*7*, *13*, *33*, *52*–*55*). This class of microrobots could enable targeted drug delivery, trigger mechanotransducive mechanisms, or serve as active tracers for the mapping of diseased tissue. Moreover, the inherent advantages of these ultrasound-driven microrobots, including low-cost actuation equipment, rapid swimming speeds, and the biocompatible nature of their acoustic actuation, underscore their potential to advance localized therapeutic and diagnostic interventions (*4*, *32*, *43*).

In this context, we anticipate that acoustic microrobotics and acoustofluidic devices will further expand their impact in the field in the coming decade. By leveraging combinations of the unique features of acoustics—such as high actuation forces at the microscale, simple frequency-selective actuation, and high biocompatibility—as well as its widespread use in clinical settings, acoustic manipulation systems hold great potential for biomedical applications (*33*, *56*–*58*). For instance, recent studies have shown that bubble-based acoustic microrobots can achieve impressive swimming speeds of up to 350 mm/s (≈17,500 body lengths per second), making them well suited to withstand the high shear stress levels experienced in physiologically relevant environments (*32*). While challenges remain, such as the temporal bubble instability caused by rectified diffusion, recent advancements have substantially improved bubble stability, enabling these microrobots to navigate biological fluids, achieve three-dimensional (3D) steerability, and even manipulate single particles (*4*, *33*). Further recent developments, including acoustic bubble-based tweezers and sharp-edged end-effectors, highlight the potential to expand acoustofluidic principles beyond drug delivery and lab-on-chip devices, making them more accessible for medical tools as well as automated high-throughput processes (*59*–*61*). Growing interest from the tissue engineering community in acoustically assisted 3D printing has driven the use of acoustic waves to precisely manipulate and assemble cells, enabling the creation of complex biostructures with high spatial accuracy (*62*–*64*). Here, acoustofluidic patterning offers key advantages, including noncontact manipulation, high biocompatibility, and precise in-scaffold control, making it a versatile tool for constructing multilayered structures with high-throughput capabilities (*65*, *66*).

Ultimately, the integration of real-time Doppler ultrasound imaging, such as in concepts introduced in this study, advances microrobotic research toward active drug delivery in a patient-friendly, minimally invasive manner, contributing to the progress of precision medicine (*67*, *68*). These applications could include the treatment of bladder cancer through localized drug delivery, addressing stomach ulcers by precisely targeting ulcerated areas with therapeutic agents, and managing inflammatory bowel disease, such as Crohn’s disease or ulcerative colitis, by delivering anti-inflammatory drugs directly to affected intestinal tissues (*52*). However, these advancements not only can increase treatment precision but also broaden the applications of microrobotics in health care. Our findings have the potential to influence future research and innovation in diagnostic disease mapping, localized neuromodulation, and real-time tissue monitoring. Conclusively, this study reports a method by which ultrasound imaging can overcome its resolution deficit at the microscale and enable effective real-time observation of next-generation microrobotic therapeutics and diagnostics.

## MATERIALS AND METHODS

### Microrobot fabrication

The bubble-based microrobots were 3D printed using a two-photon polymerization system (Photonic Professional GT, Nanoscribe GmbH, Karlsruhe, Germany) with a 25× objective (numerical aperture, 0.8). We printed arrays of microrobots on indium tin oxide–coated glass substrates using the biocompatible IP-S photoresin (Nanoscribe GmbH, Karlsruhe, Germany), enabling high feature size-resolution printing. After 3D printing, the microrobots were developed in propylene glycol monomethyl ether acetate (Sigma-Aldrich) for 20 min and rinsed in isopropyl alcohol for another 5 min. To ensure hydrophobic cavities, we dried the microrobots for 20 min at 85°C and then performed a silanization (1*H*,1*H*,2*H*,2*H* perfluorooctyl-trichlorosilane, Sigma-Aldrich) process in a vacuum chamber for 45 to 60 min.

### Ultrasound phantom preparation

To prepare agar gel, 8 g of agar (agar powder, Migros, Switzerland) is mixed with 1.5 dl of boiling water while ensuring continuous stirring. This stirring process is sustained for 3 min to ensure proper solidification of the agar gel. Subsequently, a well-mixed agar solution is carefully poured into the 3D-printed mold, which has been crafted using a 3D filament printer (ENDER 3 V2). After molding, the agar gel is cooled down in a refrigerator at 5°C for a minimum of 1 hour.

### Experimental setup

The setup is assembled as follows: First, microrobots are positioned in cross formation on a PDMS spin-coated ( duration,1min ; revolutions per minute, $1000\;{\text{min}}^{-1}$ ) glass slide ( 38by75by1mm ) using an optical fiber (AFS 100/110/130T, Fiberguide Industries Inc.) under careful attention on an optical inverted microscope. Next, the agar phantom, prepared previously, is gently released from the mold, ensuring that the chamber faces upward. The upside-down lying chamber is then filled with DI water. The glass slide, bearing the microrobots facing downward, is slowly lowered over the chamber from one side to the other, with a slow approach to reduce the risk of microrobots being flushed away and to prevent air bubble formation between the chamber and the glass slide. Subsequently, the phantom with the glass slide is carefully flipped by 180° so that the phantom is now positioned on the glass substrate. To ensure optimal coupling between the manipulation probe, comprising a piezoelectric transducer (Murata, 7BB-27-4L0) attached to a conventional microscope glass slide ( 25by75by1mm ), and the phantom, as well as between the imaging probe and the phantom, ultrasound coupling gel (K-Y gel, medical sterile) is applied in both cases. By mounting this assembly on an inverted microscope, with optical access from below and ultrasound imaging from the side, optical verification of what is observed under ultrasound is enabled.

### Ex vivo experiment

For these experiments, we used euthanized female 129S1/SvImJ mice. For catheter preparation, polyethylene (PE-10) tubing was cut into 13-cm segments and sterilized by immersion in 70% ethanol. Microrobots (~400 per injection) were suspended in 200 μl of sterile water immediately before use. For nonsurvival procedures, mice were euthanized via carbon dioxide inhalation followed by pneumothorax, with complete cessation of vital signs confirmed before proceeding. The lower abdomen was shaved to facilitate ultrasound coupling, and the perineal region was disinfected with 70% ethanol. Catheterization was performed by attaching sterile PE-10 tubing to a 30G blunted needle and syringe, lubricating the tubing with lidocaine gel, and gently inserting it into the female urethra to a depth of 1.5 to 2 cm, halting upon encountering resistance. The microrobot suspension was administered slowly at a rate of 30 to 50 μl/min using a Hamilton syringe, with a total injection volume not exceeding 100 μl. Following injection, the catheter was maintained in position for 1 to 2 min to promote uniform distribution within the bladder. Mice were then positioned supine on an inverted manipulation probe (piezoelectric transducer attached to the microscope glass slide), and ultrasound gel was applied to the shaved abdominal surface. Microrobots were activated with f=99.5 to 101.5kHz , and real-time CFM-mode ultrasound imaging was used to detect microrobots within the bladder cavity.

### Imaging and data analysis

For experiments, a Zeiss Axiovert 200M inverted microscope was used and equipped with a high-speed camera (CHRONOS 1.4, Kron Technologies). Recorded data were analyzed using software such as ImageJ and MATLAB. The ultrasound imaging system used in all experiments is a portable Color Doppler ultrasound scanner (Sonoscape E2, imaging frequencies $f_{\text{US}}=4.0\;\text{to}\;16.0\;\text{MHz}$ ) equipped with a linear array imaging probe (Sonoscape, L741).
