# Supplementary material for: Real-time color flow mapping of ultrasound microrobots
Source: Sci Adv. 2025 Jul 18;11(29):eadt8887. doi: 10.1126/sciadv.adt8887 (PMC12273784; doi:10.1126/sciadv.adt8887)
Supplement: Supplementary file 1 — Supplementary Text Figs. S1 to S5 Table S1 Legends for movies S1 to S8 [file sciadv.adt8887_sm.pdf]

Science Advances
AAAS

Supplementary Materials for

Real-time color flow mapping of ultrasound microrobots

Cornel Dillinger *et al.*

Corresponding author: Daniel Ahmed, dahmed@ethz.ch

Sci. Adv. **11**, eadt8887 (2025)
DOI: 10.1126/sciadv.adt8887

The PDF file includes:

Supplementary Text
Figs. S1 to S5
Table S1
Legends for movies S1 to S8

Other Supplementary Material for this manuscript includes the following:

Movies S1 to S8

Supplementary Information

Free bubble resonance frequency:
The natural resonance frequency of a free microbubble under adiabatic conditions, neglecting surface tension effects, and matching the size of the microrobot's cavity in this study, can be computed as (*37*),

$$f_{res} = \frac{1}{2\pi r_0}\sqrt{\frac{3\gamma p_0}{\rho}} \approx 100.0 \text{ kHz},$$

where $r_0 \approx 32.8325$ µm represents the radius of the microbubble and $\gamma = 1.4$, $p_0 = 1\ atm$, and $\rho = 1000.0\ \frac{kg}{m^3}$ are the adiabatic index, the atmospheric pressure, and the density of the surrounding fluid.

Supplementary Figures & Tables

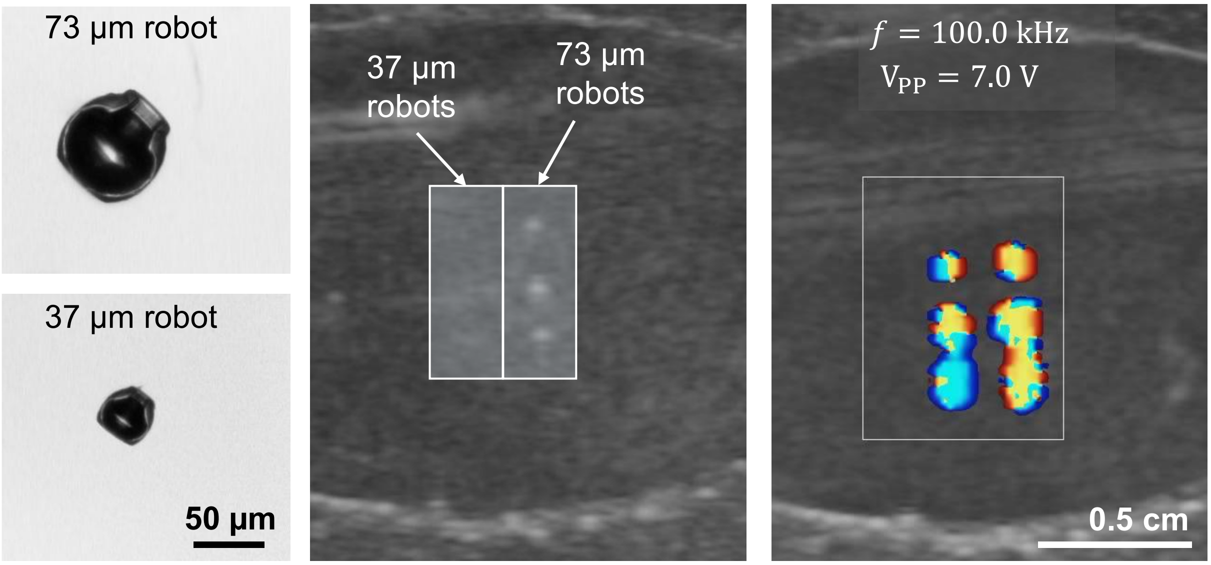

Fig. S1. CFM-mode imaging of various size microrobots. Microrobots with diameter $d_{small} \approx 37.0$ µm and $d_{big} \approx 73.0$ µm were simultaneously imaged using CFM-mode and acoustic stimulation with $f = 100.0$ kHz.

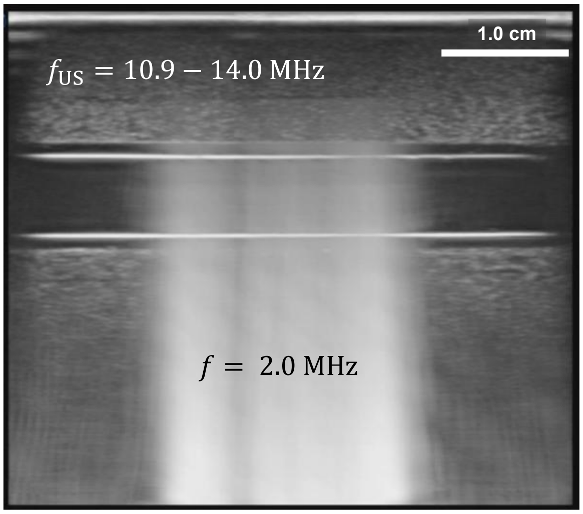

Fig. S2. Interference of simultaneous ultrasonic stimulation and imaging. When a stimulating ultrasonic field of $f = 2.0$ MHz (sine wave actuation of piezoelectric transducer) is applied to a vascularized ultrasound phantom, the field interfered with the ultrasonic imaging ($f_{US} = 10.9 -$ 14.0 MHz), resulting in brightness fluctuations in the imaging system. These disturbances originate not only from interfering acoustic fields but also from insufficient electrical shielding of our manipulation probe, leading to signal interferences when the probe is actuated but not in contact with the ultrasound phantom.

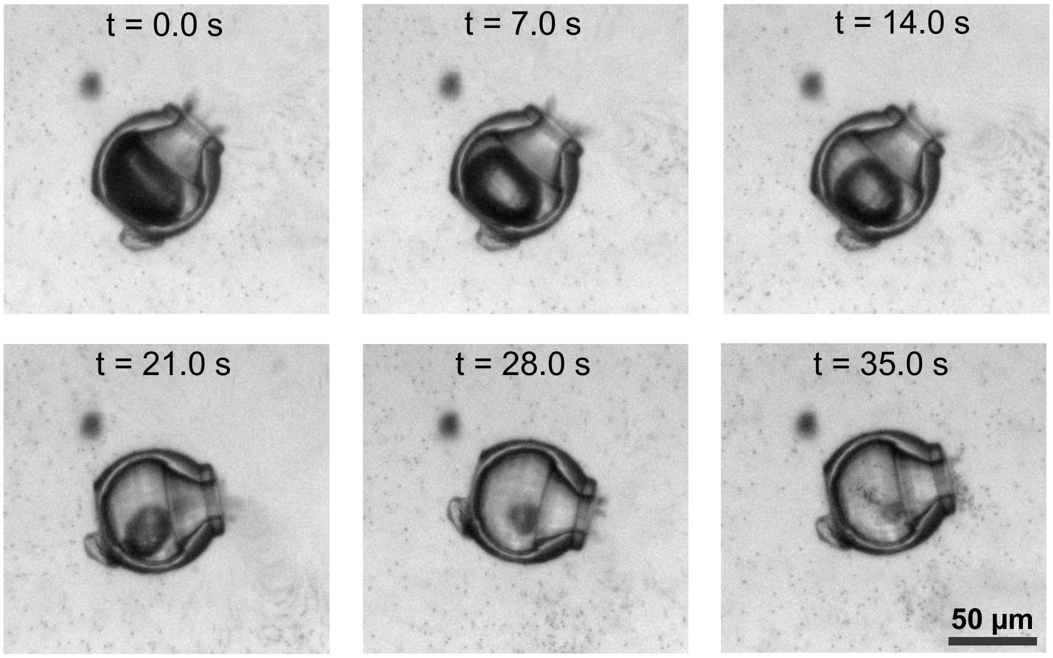

Fig. S3. Bubble collapse under high acoustic field amplitude. Entrapped microbubbles often collapsed when exposed to applied amplitudes greater than $V_{pp} \approx 50.0$ V (here: $f = 101.5$ kHz and $V_{pp} = 58.5$ V).

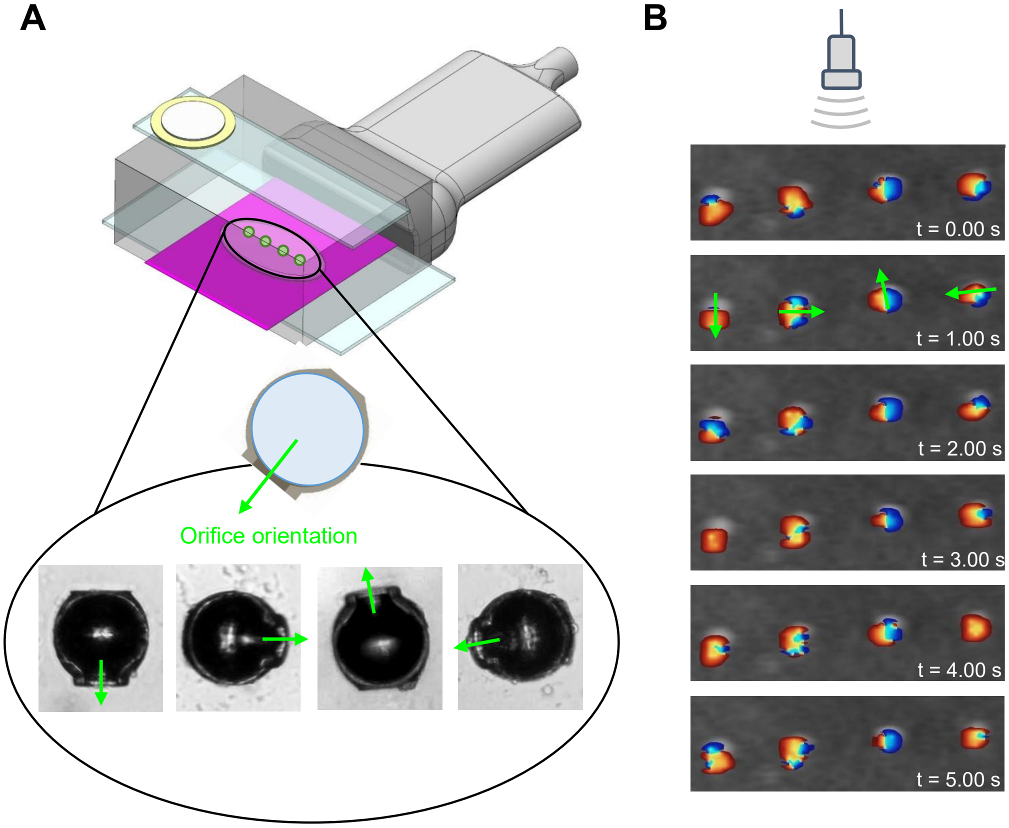

Fig. S4. Microrobot orientation independency for detection. (**A**) Four microrobots positioned with different orifice orientations were placed in the ultrasound phantom. (**B**) Under stimulation with an acoustic field of $f = 103.6$ kHz and $V_{pp} = 42.0$ V, no characteristic orientation-dependent signal was detected in CFM-mode ultrasound imaging.

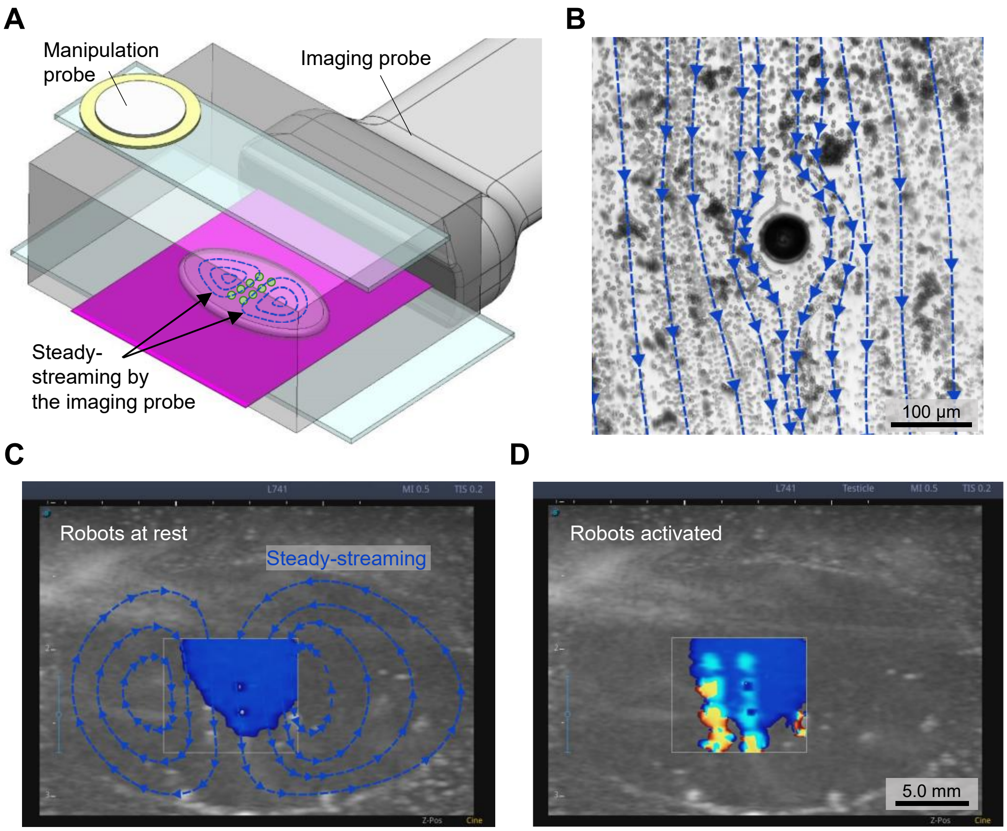

Fig. S5. Visualization of microrobots under Doppler-detected steady-streaming.(**A**) Schematic of steady-streaming, i.e., Eckart streaming (*47*), within the elliptical chamber of the ultrasound phantom, generated by the imaging probe. To visualize the steady-streaming, 6 µm flow tracer particles were added to the DI-water solution. In the optical Z-stack image of (**B**), the flow field around one of the eight positioned microrobots is depicted. (**C**) Due to the flow tracers immersed in the DI-water, CFM-mode ultrasound imaging ($f_{\mathrm{PRF}} = 1.2\,\mathrm{kHz}$) of the microrobots at rest resulted in Doppler-detected steady-streaming covering the microrobots. (**D**) Activated by the manipulation probe ($f = 100.0\,\mathrm{kHz}, \mathrm{V_{pp}} = 3.5\,\mathrm{V}$), the microrobots became clearly visible using CFM-mode imaging, even amidst the Doppler-detected steady streaming.

Acoustic field f [kHz]	100.0	100.0	100.0	100.0	100.0	100.0	100.0	100.0	100.0	100.0
Pulse Repetition Frequency f_{PRF} [kHz]	10.0	8.8	8.0	7.0	6.7	6.0	5.7	5.0	4.4	4.0
$\frac{f}{f_{\text{PRF}}}$	10.0	11.364	12.5	14.286	14.925	16.667	17.544	20.0	22.727	25.0
CFM signal	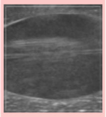	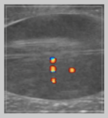	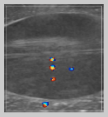	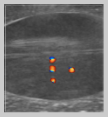	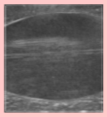	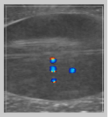	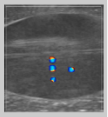	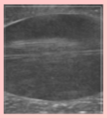	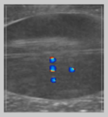	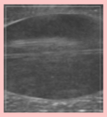

Acoustic field f [kHz]	100.0	100.0	100.0	100.0	100.0	100.0	100.0	100.0	100.0	100.0
Pulse Repetition Frequency f_{PRF} [kHz]	3.2	3.0	2.5	2.0	1.8	1.5	1.2	1.0	0.8	0.5
$\frac{f}{f_{\text{PRF}}}$	31.25	33.333	40.0	50.0	55.556	66.667	83.334	100.0	125.0	200.0
CFM signal	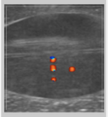	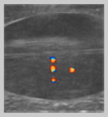	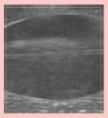	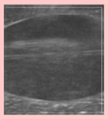	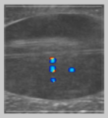	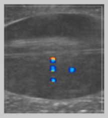	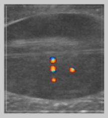	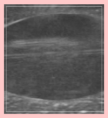	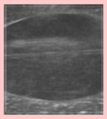	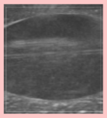

Table S1. Dependence of CFM signal on the interference between the stimulating acoustic field and Pulse Repetition Frequency (PRF).

When the stimulation frequency of the acoustic field divided by the Pulse Repetition Frequency ($\frac{f}{f_{PRF}}$), in the ultrasound imaging system resulted in an integer (except when f_{PRF} = 6.7 kHz), the CFM-mode failed to detect the activated, oscillating microrobots. In this experiment, the robot positioned to the left within the cross formation remained non-responsive (no microbubble entrapped).

Movie S1.
Frequency sweep experiment: The response of a microbubble entrapped within a microrobot to an acoustic frequency sweep over the range of $f = 97.0 - 105.0$ kHz ($V_{PP} = 7.0$ V) is captured using a CCD camera and an ultrasound imaging system operating in CFM-mode.

Movie S2.
Power sweep experiment: Microrobots exposed to an acoustic field at $f = 101.0$ kHz with voltage peak-to-peak (V_{PP}) power amplitudes ranging from $V_{PP} = 0.7 - 14.0$ V are captured using a CCD camera and an ultrasound imaging system operating in CFM-mode.

Movie S3.
Variable imaging plane: At $\theta_{Probe} = 60°$, with improved proficiency in handling the imaging probe, it is shown that varying the imaging plane to display various rows of microrobots is feasible. Acoustic stimulation parameters: $f = 101.0$ kHz and $V_{PP} = 27.3$ V.

Movie S4.
Deep tissue experiment: Five microrobots are visualized in real-time at an ultrasound phantom depth of 10 cm. Acoustic stimulation parameters: $f = 101.0$ kHz and $V_{PP} = 21.0$ V.

Movie S5.
Real-time motion experiment 1: The microrobot's spherical-like motion was recorded simultaneously using a CCD camera and the ultrasound imaging system in CFM-mode. Acoustic stimulation parameters: $f = 101.5$ kHz and $V_{PP} = 27.3$ V.

Movie S6.
Real-time motion experiment 2: The microrobot's linear-like motion was recorded simultaneously using a CCD camera and the ultrasound imaging system in CFM-mode. Acoustic stimulation parameters: $f = 101.5$ kHz and $V_{PP} = 27.3$ V.

Movie S7.
Drug-delivery mechanism: Acoustic microbubble streaming (1 µm-flow tracers) directed toward the wall can be utilized to perfuse dissolved drugs to the cavity's wall. Acoustic stimulation parameters: $f = 101.0$ kHz and $V_{PP} = 21.0$ V.

Movie S8.
Ex vivo experiment: Microrobots injected into an *ex vivo* mouse bladder model are manipulated ($f = 99.5 - 101.5$ kHz and $V_{PP} = 45.0$ V) and visualized using CFM-mode ultrasound imaging.
